# Effects of Daily Zinc Alone or in Combination with Other Nutrient Supplements on the Risk of Malaria Parasitaemia: A Systematic Review and Meta-Analysis of Randomised Controlled Trials

**DOI:** 10.3390/nu15132855

**Published:** 2023-06-23

**Authors:** Manas Kotepui, Polrat Wilairatana, Wanida Mala, Kwuntida Uthaisar Kotepui, Frederick Ramirez Masangkay, Kinley Wangdi

**Affiliations:** 1 Medical Technology, School of Allied Health Sciences, Walailak University, Tha Sala, Nakhon Si Thammarat 80160, Thailand; 2 Department of Clinical Tropical Medicine, Faculty of Tropical Medicine, Mahidol University, Bangkok 10400, Thailand; 3 Department of Medical Technology, University of Santo Tomas, Manila 1008, Philippines; 4 Department of Global Health, National Centre for Epidemiology and Population Health, College of Health and Medicine, Australian National University, Canberra, ACT 2601, Australia

**Keywords:** zinc, micronutrient, malaria, Plasmodium, food supplementation, micronutrients

## Abstract

Zinc supplementation has been explored as a potential intervention to reduce the risk of malaria parasitaemia in randomised controlled trials (RCTs). However, inconsistent evidence has been obtained regarding the efficacy of zinc supplementation in the context of malaria prevention. This systematic review was implemented to survey the existing literature to determine the effects of the daily oral administration of zinc, either alone or in combination with other nutrient supplements, on the risk of malaria parasitaemia. The systematic review was prospectively registered in the PROSPERO database CRD42023424345 and followed PRISMA protocols. A comprehensive search was conducted across multiple databases, including Embase, MEDLINE, Ovid, PubMed, Scopus, ProQuest, and Google Scholar, from their inception until 6 May 2023. The risk of bias in RCTs was assessed using the Cochrane Risk of Bias Tool 2 (RoB 2). The effect sizes, represented as risk ratios (RRs) with 95% confidence intervals (CIs), were standardised by transforming them into log RRs and then pooling them using a fixed-effects or random-effects model depending on the heterogeneity across studies. Comparisons were made between individuals who received zinc alone or zinc in combination with other micronutrient supplements and those who did not receive zinc. A total of 1339 articles were identified through the database searches, and after the screening and selection process, 10 studies were included in the final synthesis. The meta-analysis revealed that zinc supplementation alone did not significantly affect the risk of malaria parasitaemia compared with placebo (*p* = 0.30, log RR = 0.05, 95% CI: −0.05–0.15, *I*^2^ = 0.00%, with 566 malaria cases in the zinc intake group and 521 malaria cases in the placebo group). However, the analysis demonstrated a borderline significant effect of zinc supplementation in combination with other micronutrients on the risk of malaria parasitaemia compared with placebo (*p* = 0.05, log RR = 1.31, 95% CI: 0.03–2.59, *I*^2^ = 99.22%, with 8904 malaria cases in the zinc intake group and 522 malaria cases in the placebo group). The findings of this systematic review indicate that zinc supplementation, either alone or combined with the supplementation of other micronutrients such as vitamin A, iron, or multiple nutrients, does not significantly alter the risk of malaria parasitaemia. Further research with larger sample sizes is warranted to explore the potential effects of multi-nutrient supplementation and to identify more specific micronutrients and additional factors associated with the risk of malaria, rather than just zinc alone, among individuals in different malaria-endemic areas.

## 1. Introduction

Malaria is a potentially life-threatening infectious disease caused by parasites of the genus *Plasmodium*. The disease is primarily transmitted to humans through the bites of infected female *Anopheles* mosquitoes [1]. Several different species of *Plasmodium* parasites can cause malaria in humans, with *Plasmodium falciparum* being the deadliest and most prevalent [2,3]. However, other species, such as *P. vivax*, *P. ovale*, *P. malariae*, and *P. knowlesi*, can also cause malaria, although cases involving their infection tend to be less severe [2]. There is a complex interplay between malaria and the immune system. The immune system recognises the presence of the parasite and mounts an immune response against it [4,5]. However, such an infection may impair the development and function of immune cells, leading to immune dysfunction and increased susceptibility to severe disease or other infections [6,7]. In addition, malaria infection contributes to an increased production of reactive oxygen species (ROS) which help to eliminate the parasite; however, excessive amounts of ROS can mediate extensive inflammation and damage to host cells, which may contribute to severe disease [8,9].

Zinc is an essential mineral for maintaining human health that is involved in numerous physiological processes including immunity, wound healing, growth and development, taste and smell sensations, reproductive health, cognitive function, and antioxidant activities [10,11,12]. Zinc deficiency can lead to delayed growth and development, reproductive and fertility issues, impaired immune function, loss of appetite, weight loss, impaired physical function and frailty, and skin and hair problems [13,14,15]. It has also been reported that malaria can contribute to zinc deficiency through increased inflammation and oxidative stress [16] and that such a deficiency can increase susceptibility to malaria [17,18]. It has been reported that zinc supplementation as an adjunct therapy to standard antimalarial treatment can improve haematological parameters and reduce the mortality of mice [19]. However, in human participants, some studies reported no significant effect of zinc on the treatment of acute uncomplicated malaria [20].

Despite significant advances in malaria prevention and treatment, the disease continues to pose a significant threat to human health globally, particularly in malaria-endemic regions. Zinc, an essential mineral with diverse physiological functions, has been implicated in the immune response and is hypothesised to play a role in modulating the risk of malaria parasitaemia. However, previous studies yielded inconsistent results regarding the effectiveness of zinc supplementation in relation to malaria treatment and prevention, highlighting the need for a systematic review to synthesise the available evidence. This systematic review was thus established to investigate the effect of zinc supplementation or supplementation with zinc plus other micronutrients on the risk of malaria parasitaemia in individuals living in malaria-endemic areas. By critically evaluating the existing literature, this review is intended to provide a comprehensive understanding of the potential impact of zinc supplementation on malaria outcomes and should contribute to the development of effective preventive strategies.

## 2. Methods

### 2.1. Registration and Reporting of Systematic Review

The protocol of this systematic review was registered in the International Prospective Register of Systematic Reviews (PROSPERO, registration number: CRD42023424345). The systematic review adhered to the Preferred Reporting Items for Systematic Reviews and Meta-Analyses (PRISMA) guidelines for reporting systematic reviews [21], ensuring transparent and comprehensive reporting of the review process.

### 2.2. Research Questions

The Population, Intervention, Comparison, and Outcome (PICO) framework was applied to the research question as follows: P, individuals living in malaria-endemic areas; I, zinc supplementation or supplementation with zinc plus other micronutrients; C: no intervention or placebo or potentially comparing different types of interventions; and O, risk of malaria parasitaemia. The research question for developing this systematic review was as follows: “In individuals living in malaria-endemic areas, does zinc supplementation or supplementation with zinc plus other micronutrients (compared with placebo) affect the risk of malaria parasitaemia”?

### 2.3. Eligibility Criteria

Eligible studies included participants from malaria-endemic areas who received either zinc supplementation alone or in combination with other micronutrients in comparison to a placebo group. The outcome of interest was the risk of malaria parasitaemia. In terms of the study design, the studies had to be randomised controlled trials (RCT) or controlled clinical trials. Literature such as reviews, systematic reviews, case reports, and case series were excluded from the present review. There was no language restriction.

### 2.4. Search Strategy

A combination of keywords (including relevant synonyms or alternative terms) and Boolean operators was applied in the search strategy as follows: ‘Zinc” AND (“Malaria” OR “*Plasmodium*” OR “*Plasmodium* Infection” OR “Remittent Fever” OR “Marsh Fever” OR “Paludism”) AND (“Clinical Trials” OR “Randomised Trials” OR “Randomised Controlled Trials” OR “Controlled Clinical Trials”). The search strategy was adapted based on the specific databases or search engines (Table S1). A search in Google Scholar was also conducted to identify relevant scholarly articles.

### 2.5. Study Selection

After articles identified from databases were imported into Endnote software (version 20.0; Clarivate Analytics, Philadelphia, PA, USA), duplicates were removed. Two authors (M.K. and K.U.K.) conducted the study selection independently by applying the inclusion/exclusion criteria to the remaining articles. The processes involved screening the titles/abstracts and reviewing the full texts of the articles. Any discrepancies or uncertainties were resolved through discussion.

### 2.6. Data Extraction

The extraction of relevant data from selected studies was conducted using a structured data extraction form. Common elements included were as follows: study details (first author, year of publication, and the year in which the study was conducted), participant characteristics (number, age, sex, nationality, and status of *Plasmodium* infection), intervention details (supplementations with zinc and other micronutrients or placebo), outcome measures (risk of malaria), and methods for identifying malaria and measurement of zinc levels. Data were independently extracted from each included study by two independent authors (M.K. and K.U.K.). The extracted data were cross-checked and evaluated for accuracy and consistency by a third author (P.W.).

### 2.7. Risk of Bias

The Cochrane Risk of Bias Tool 2 (RoB 2) was used for assessing the risk of bias in RCTs [22]. The RoB 2 uses five domains to assess the risk of bias in studies, including bias arising from the randomisation process, bias resulting from deviations from intended interventions, bias resulting from missing outcome data, bias in the measurement of the outcome, and bias in the selection of the reported result. The classifications of “low”, “some concerns”, and “high” were used to assess the overall risk of bias for each domain. Regarding the overall judgment of the risk of bias for each study, this was set as “Low risk of bias” if the study was judged to be at a low risk of bias for all domains, “Some concerns” if the study was judged to be associated with some concerns in at least one domain, and “High risk of bias” if the study was judged to be associated with a high risk of bias in at least one domain [22] (Table S3).

### 2.8. Statistical Analysis

The effect sizes (risk ratios (RRs)) were standardised across studies by transforming the effect sizes into log RRs. The weight of each study was assigned based on sample size by inversing the variance (fixed-effects model) if there was no significant heterogeneity of the effect sizes across studies. Meanwhile, a random-effects model was used to pool the effect size if there was significant heterogeneity of the effect sizes across studies. The significance of heterogeneity was assessed using Cochran’s Q test and the heterogeneity was quantified using the *I*^2^ statistic. A significant Cochran’s Q test result (*p* < 0.10) indicated that the observed variation in effect sizes was greater than what would be expected by chance alone. *I*^2^ values of 25%, 50%, and 75% indicate low, moderate, and high heterogeneity, respectively [23]. A subgroup analysis and a meta-regression analysis were conducted to explore potential sources of significant heterogeneity in different study-level characteristics (age, nationality, *Plasmodium* species, and risk of bias). A sensitivity analysis was performed to evaluate the robustness of the results by excluding one study at a time and rerunning the meta-analysis [24]. The presence of publication bias was assessed using funnel plots and Egger’s test to detect asymmetry in the distribution of effect sizes if the number of studies included in the meta-analysis exceeded 10 [25].

## 3. Results

### 3.1. Search Results

**Figure 1:**
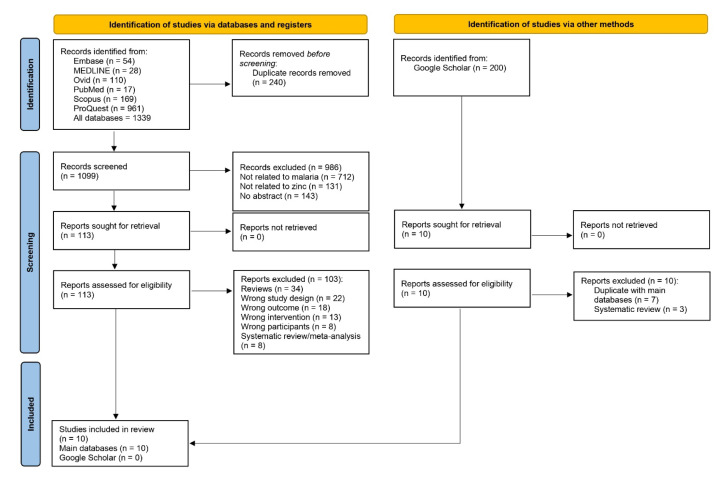
Study flow diagram.

A total of 1339 articles were identified from various databases, including Embase (*n* = 54), MEDLINE (*n* = 28), Ovid (*n* = 110), PubMed (*n* = 17), Scopus (*n* = 169), and ProQuest (*n* = 961). After removing 240 duplicate records, 1099 articles were screened. Among these, 986 articles were excluded due to being unrelated to malaria (*n* = 712), unrelated to zinc (*n* = 131), or lacking an abstract (*n* = 143). A total of 113 reports were retrieved and assessed for eligibility, and ultimately, 10 studies met the criteria and were included for synthesis [26,27,28,29,30,31,32,33,34,35]. None of the articles obtained from Google Scholar met the eligibility criteria (Figure 1).

### 3.2. Characteristics of the Included Studies

The reports on the ten studies included in this review were published between 2000 and 2017. The studies were conducted in Burkina Faso (30%) [26,28,35], Tanzania (30%) [27,32,34], Ghana (20%) [29,31], Papua New Guinea (10%) [33], and Peru (10%) [30]. Eight studies (80%) enrolled children [26,28,29,30,32,33,34,35], while two studies (20%) enrolled pregnant women [27,31]. The participants in the studies took zinc or other nutrient supplements orally on a daily basis, with variations in the durations of intake and follow-up periods. Some studies focused on detecting *P. falciparum* infection alone, while others assessed infections with both *P. falciparum* and other *Plasmodium* species (Table 1). Detailed information on the studies is presented in Table S2.

### 3.3. Risk of Bias

Five included studies presented a low risk of bias [29,30,32,33,34], four had some concerns [27,28,31,35], and one was considered to have a high risk of bias [26]. The randomisation process was determined to have a low risk of bias in all studies. For deviations from the intended interventions (the effect of assignment to intervention), domains 2.1 to 2.5, all studies had a low risk of bias (100%). For deviations from the intended interventions (the effect of assignment to intervention), domains 2.6 to 2.7, six studies had a low risk of bias (60%), while four studies had some concerns (40%). Regarding deviations from the intended interventions (the effect of adhering to intervention), eight studies had a low risk of bias (80%), while two studies had a high risk of bias or some concerns (20%). There were low risks of bias regarding missing outcome data in nine studies (90%), while one study had some concerns (10%). All studies included in the systematic review had low risks of bias in terms of outcome measurement and result selection (100%) (Table S3).

### 3.4. Effect of Zinc Alone on Risk of Malaria Parasitaemia

**Figure 2:**
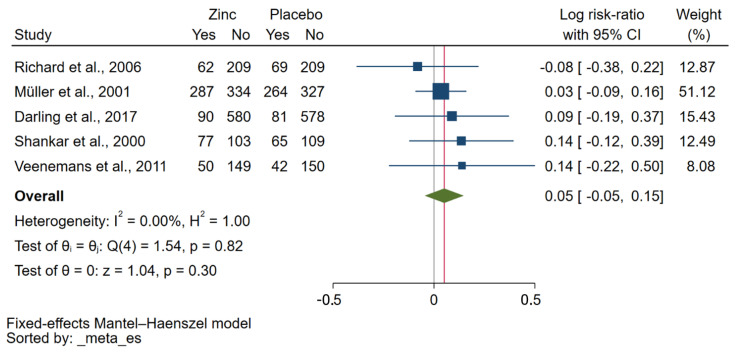
A forest plot shows the meta-analysis of five studies comparing the zinc and placebo groups. The effect of zinc supplementation on the risk of malaria parasitaemia at the endpoint was demonstrated as the log RR. Symbol explanations: Blue-squared boxes show effect estimates; Blue horizontal lines show 95% CI, Green diamond indicates pooled effect estimate. Abbreviation: CI, confidence interval [27,28,30,33,34].

Qualitative synthesis showed that zinc supplementation did not reduce the incidence of malaria parasitaemia, as shown by six RCTs [26,27,28,30,33,34]. A meta-analysis of five studies [27,28,30,33,34] comparing the zinc and placebo groups found no significant effect of zinc on the risk of malaria parasitaemia at the endpoint (*p*: 0.30, log RR: 0.05, 95% CI: −0.05–0.15, *I*^2^: 0.00%, 566 malaria cases in the zinc group/521 malaria cases in the placebo group, Figure 2). Similarly, when considering *P. falciparum* infection alone, a meta-analysis also showed no significant effect of zinc on the risk of *P. falciparum* parasitaemia at the endpoint (*p*: 0.26, log RR: 0.05, 95% CI: −0.05–0.15, *I*^2^: 0.00%, 437 malaria cases in the zinc group/395 malaria cases in the placebo group, Supplementary Figure S1).

### 3.5. Effect of Zinc Plus Other Micronutrients on Risk of Malaria Parasitaemia

**Figure 3:**
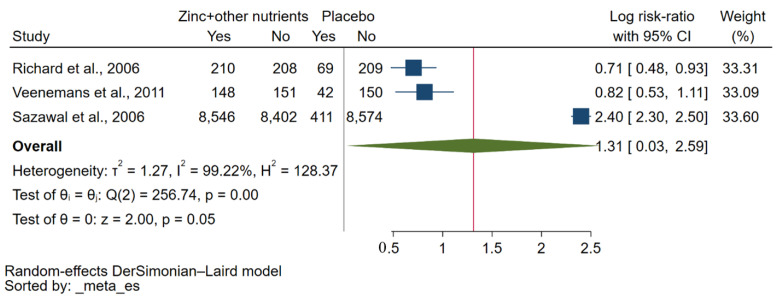
A forest plot shows a meta-analysis of three studies comparing zinc plus other micronutrients with placebo regarding the risk of malaria parasitaemia at the endpoint. The effect of supplementation with zinc plus other micronutrients on the risk of malaria parasitaemia at the endpoint was demonstrated as log RR. Symbol explanations: Blue-squared boxes show effect estimates; Blue horizontal lines show 95% CI, Green diamond indicates pooled effect estimate. Abbreviation: CI, confidence interval [30,32,34].

A qualitative synthesis showed that zinc plus vitamin A supplementation had been reported to reduce the incidence of malaria parasitaemia, as shown by two studies [29,35]. However, no significant effect of zinc plus iron supplementation on the risk of malaria was demonstrated by two studies [30,31]. Supplementation with zinc plus multiple other nutrients did not influence malaria rates, as demonstrated by Veenemans et al. [34]. A meta-analysis of three studies [30,32,34] comparing zinc plus other micronutrients with placebo showed a borderline significant effect on the risk of malaria parasitaemia at the endpoint (*p*: 0.05, log RR: 1.31, 95% CI: 0.03–2.59, *I*^2^: 99.22%, 8904 malaria cases in the zinc plus other micronutrients group/522 malaria cases in the placebo group, Figure 3). However, when considering *P. falciparum* infection alone, there was no significant effect of zinc plus other micronutrients on the risk of *P. falciparum* parasitaemia at the endpoint compared with placebo (*p*: 0.62, log RR: −0.09, 95% CI: −0.45–0.27, *I*^2^: 66.19%, 548 malaria cases in the zinc plus other micronutrients group/473 malaria cases in the placebo group, Supplementary Figure S2).

**Figure 4:**
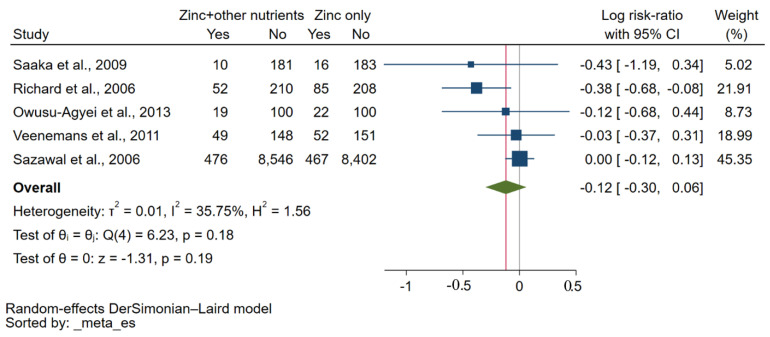
A forest plot shows a meta-analysis of five studies comparing other micronutrients without zinc to zinc only. The effect of other micronutrients without zinc on the risk of malaria parasitaemia at the endpoint was demonstrated as the log RR. Symbol explanations: Blue-squared boxes show effect estimates; Blue horizontal lines show 95% CI, Green diamond indicates pooled effect estimate. Abbreviation: CI, confidence interval [29,30,31,32,34].

**Figure 5:**
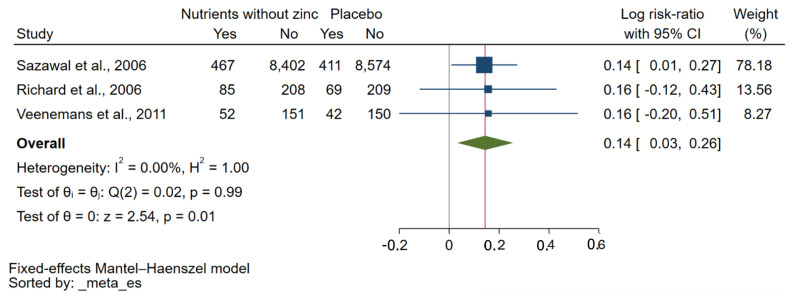
A forest plot shows a meta-analysis of three studies comparing other micronutrients without zinc to placebo. The effect of other micronutrients without zinc on the risk of malaria parasitaemia at the endpoint was demonstrated as log RR. Symbol explanations: Blue-squared boxes show effect estimates; Blue horizontal lines show 95% CI, Green diamond indicates pooled effect estimate. Abbreviation: CI, confidence interval [30,32,34].

A meta-analysis of five studies [29,30,31,32,34] comparing zinc plus other micronutrients with zinc alone showed no significant effect on the risk of malaria parasitaemia at the endpoint (*p*: 0.19, log RR: −0.12, 95% CI: −0.30–0.06, *I*^2^: 35.75%, 606 malaria cases in the zinc plus other micronutrients group/642 malaria cases in the zinc alone group, Figure 4). Similarly, when considering *P. falciparum* infection alone, there was no significant effect of zinc plus other micronutrients on the risk of malaria parasitaemia at the endpoint compared with zinc alone (*p*: 0.86, log RR: −0.01, 95% CI: −0.13–0.11, *I*^2^: 0.00%, 497 malaria cases in the zinc plus other micronutrients group/495 malaria cases in the zinc alone group, Supplementary Figure S3). A meta-analysis of three studies [30,32,34] comparing other micronutrients without zinc to placebo showed a significant effect on the risk of malaria parasitaemia at the endpoint (*p*: 0.01, log RR: 0.14, 95% CI: 0.03–0.26, *I*^2^: 0.00%, 604 malaria cases in the other micronutrients without zinc group/522 malaria cases in the placebo group, Figure 5).

### 3.6. Sensitivity Analysis

Using a leave-one-out meta-analysis, there was no significant effect of zinc on the risk of malaria parasitaemia at the endpoint compared with placebo in all rerun analyses (*p* > 0.05). This indicates the robustness of the meta-analysis results (Supplementary Figure S4). For *P. falciparum* infection alone, the leave-one-out meta-analysis also indicated that zinc supplementation had no substantial effect on the risk of malaria parasitaemia at the endpoint in every rerun analysis (*p* > 0.05), demonstrating the reliability of the meta-analysis results (Supplementary Figure S5).

Using the leave-one-out meta-analysis, there was no significant effect of zinc plus other micronutrients on the risk of malaria parasitaemia at the endpoint compared with placebo after removing and rerunning the analysis without the study by Veenemans et al. [34] (*p* > 0.05). However, there were significant effects of zinc plus other micronutrients on the risk of malaria parasitaemia at the endpoint compared with placebo when the meta-analysis was rerun after removing each of two studies [30,32] (*p* < 0.05), indicating that the meta-analysis results were not robust (Supplementary Figure S6). Considering *P. falciparum* infection alone, the leave-one-out meta-analysis showed no significant effect of zinc plus other micronutrients on the risk of malaria parasitaemia at the endpoint compared with placebo in every rerun analysis (*p* > 0.05), indicating the robustness of the meta-analysis results (Supplementary Figure S7).

Using the leave-one-out meta-analysis, there was no significant effect of zinc plus other micronutrients on the risk of malaria parasitaemia at the endpoint compared with zinc alone in four rerun analyses (*p* > 0.05). However, there were significant effects of zinc plus other micronutrients on the risk of malaria parasitaemia at the endpoint compared with zinc alone after removing and rerunning the analysis without the study by Sazawal et al. [32] (*p* < 0.05, Supplementary Figure S8), indicating that the study by Sazawal et al. [32] was an outlier in the meta-analysis. For *P. falciparum* infection alone, the leave-one-out meta-analysis showed no significant effect of zinc plus other micronutrients on the risk of malaria parasitaemia at the endpoint compared to zinc alone in every rerun analysis (*p* > 0.05), indicating the robustness of the meta-analysis results (Supplementary Figure S9).

## 4. Discussion

The meta-analysis revealed that there was no statistically significant difference between the zinc group and the placebo group in terms of malaria parasitaemia risk. Furthermore, when focusing specifically on *P. falciparum* infection, the meta-analysis results demonstrated no evidence that zinc significantly affects the risk of *P. falciparum* parasitaemia at the endpoint. The results of this study imply that the risk of malaria parasitaemia or *P. falciparum* infection is not significantly reduced by zinc supplementation. Although zinc was not associated with the risk of malaria in the meta-analysis, it has been reported to reduce *P. falciparum* episodes by 38% and parasitaemia by 38% in preschool children [33]. A systematic review and meta-analysis reported in 2013 showed no significant effect of zinc supplementation on malaria-related mortality [36]. Meanwhile, another systematic review showed that zinc with and without iron supplementation may reduce the incidence of diarrhoea but has no effect on the prevalence of respiratory infections or malaria [37]. The most recent systematic review showed a statistically significant reduction in the incidence of clinical malaria episodes among participants who took zinc supplementation, but the evidence was limited by the fact that it was based on only two studies [38]. Based on an updated systematic review and previous systematic reviews [36,37], it is worth mentioning that while zinc supplementation may not directly reduce the risk of malaria or malaria-related outcomes, it may still have other important health benefits. Maintaining adequate blood zinc levels is essential for overall health as zinc is involved in various physiological processes and plays a crucial role in immune function, growth, and development [10,11,12]. However, it should not be considered a standalone preventive measure against malaria. Supplementation with micronutrients, including zinc, is one aspect of overall health and effective immunity. While adequate nutrition is essential for a robust immune system, addressing the risk of malaria requires a multifaceted approach that includes preventive measures, a prompt diagnosis, and appropriate antimalarial treatment.

The meta-analysis of three studies [30,32,34] comparing zinc plus other micronutrients to placebo revealed interesting findings regarding the risk of malaria parasitaemia at the endpoint. However, it is important to note that this meta-analysis also reported a high degree of heterogeneity among the studies, suggesting a substantial variation in the results across the included studies. Meanwhile, when considering *P. falciparum* infection alone, the meta-analysis did not find a significant effect of zinc plus other micronutrients on the risk of *P. falciparum* parasitaemia at the endpoint compared with placebo. These findings highlight the potential effect of zinc plus other micronutrients on the risk of malaria parasitaemia, but caution is needed in their interpretation. The borderline significance and the presence of substantial heterogeneity in the overall analysis indicate the need for further investigation and consideration of additional factors. Notably, the high heterogeneity may be influenced by various factors such as differences in study design, participant characteristics, dosage, and duration of supplementation, as well as variations in the intensity of malaria transmission across study settings.

The meta-analysis examined the impact of zinc plus other micronutrients compared to zinc alone on the risk of malaria parasitaemia at the endpoint, based on five studies [29,30,31,32,34]. The analysis revealed that there was no significant effect of zinc plus other micronutrients on the risk of malaria parasitaemia compared to zinc alone. Similarly, when considering *P. falciparum* infection alone, the meta-analysis found no significant effect of zinc plus other micronutrients on the risk of malaria parasitaemia compared to zinc alone. A previous study suggested that supplementation with zinc combined with vitamin A reduced fever by 22% and clinical malaria episodes by 30.2% among African children [35]. Meanwhile, the meta-analysis of three studies [30,32,34] comparing other micronutrients without zinc to placebo revealed a significant decreased risk of malaria parasitaemia at the endpoint. These findings suggest that the administration of zinc in combination with other micronutrients does not significantly impact the risk of malaria parasitaemia compared to zinc alone. However, the analysis indicates that other micronutrients without zinc may have a significant effect in reducing the risk of malaria parasitaemia compared to placebo. Nevertheless, the supplementation of micronutrients without zinc was not the main focus of analysis in this study, which could have biased the conclusion drawn here. Further research is necessary to explore the specific micronutrients involved and their potential mechanisms of action. There is also a need for studies with larger sample sizes and standardised protocols to confirm these findings and provide a more robust understanding of the relationship between micronutrient supplementation and the risk of malaria.

Although zinc was not related to the risk of malaria parasitaemia, several RCTs have demonstrated that zinc supplementation may reduce the morbidity and mortality related to diarrhoea and pneumonia [28,39]. A possible explanation for why zinc supplementation reduces the risk of diarrhoea but not malaria is that zinc plays a crucial role in maintaining intestinal barrier function, in the regeneration and repair of the gut lining, and in regulating fluid transport in the intestines [40,41]. However, zinc supplementation alone does not directly target the parasite or provide protection against malaria infection. While zinc does play a role in the immune system, it does not have direct antimalarial properties or a significant impact on the *Plasmodium* parasite itself. Therefore, while zinc supplementation may support general immune function, it is not considered an effective standalone intervention for preventing or treating malaria.

It is important to consider the limitations of the existing evidence when interpreting the meta-analysis results. Despite numerous studies that have explored the effects of zinc supplementation in combination with other micronutrients, the evidence shows modest benefits in certain populations or settings, while other studies have reported no significant effect on the incidence or severity of malaria. Based on these findings, it can be concluded that the meta-analysis of the included studies does not provide evidence supporting a significant impact of zinc supplementation on the risk of malaria parasitaemia or *P. falciparum* infection. To draw more definitive conclusions, there is a need for additional well-designed studies with larger sample sizes and standardised protocols. Further research should also aim to identify the specific mechanisms through which zinc plus other micronutrients may influence the risk of malaria parasitaemia and assess the potential benefits and risks associated with this intervention.

## 5. Conclusions

The findings of this systematic review indicate that zinc supplementation, either alone or in combination with other micronutrients such as vitamin A, iron, or multiple nutrients, did not significantly alter the risk of malaria parasitaemia. There is a need for further research with larger sample sizes to explore the potential effects of multi-nutrient supplementation and identify more specific micronutrients and additional factors involved rather than zinc alone on the risk of malaria among individuals in different malaria-endemic areas.

## Figures and Tables

**Table 1:** Characteristics of the included studies.

Author (Year)	Country	Study Period	Participant (N)	Age Range	Qualitative Synthesis	*Plasmodium* Species	Methods for Malaria	Zinc Measurement
Becquey et al., 2016 [26]	Burkina Faso	2010–2012	Children (7641)	6–30 months	Incidences of malaria were not different across study groups. (*p* = 0.48)	NS	Microscopy/RDT	Coupled plasma optical emission spectrophotometry (VWR International)
Darling et al., 2017 [27]	Tanzania	2010–2013	Pregnant women (2005)	NS	1. Participants who received zinc had a lower risk of histopathology-positive placental malaria than those who did not receive zinc; risk ratio (RR = 0.64), 2. There was no significant tendency for risk of PCR-positive malaria between participants who received zinc and those who did not.	*P. falciparum*	Placental malaria: histopathology (PCR)	NS
Müller et al., 2001 [28]	Burkina Faso	1999	Children (709)	6–31 months	No evidence for zinc supplementation being effective against *P. falciparum* malaria.	*P. falciparum*/*P. malariae*/*P. ovale*	Microscopy	Flame atomic absorption spectrometry (Perkin-Elmer 1100 B, Germany)
Owusu-Agyei et al., 2013 [29]	Ghana	2009	Children (200)	6–24 months	The number of children who were diagnosed with uncomplicated malaria in the intervention group was 27% significantly lower compared with the children in the control group (*p* = 0.03).	NS	Microscopy	Atomic Absorption Spectrophotometer AA-6300 (P/N 206-51800) Kyoto, Japan.
Richard et al., 2006 [30]	Peru	1998	Children (855)	0.5–15 years	Zinc and iron plus zinc did not statistically significantly affect *P. vivax* incidence in all children (*p* > 0.36).	*P. falciparum*/*P. vivax*	Microscopy	Atomic absorption spectrophotometry
Saaka et al., 2009 [31]	Ghana	2005–2006	Pregnant women	16–44 years	At 34-36 weeks gestation, malaria parasitaemia, though not significant, was less frequent in the iron–zinc-supplemented group compared to the iron group (adjusted OR 0.60; 95% CI: 0.25–1.44).	*P. falciparum*	Microscopy	Flame atomic absorption spectrophotometry
Sazawal et al., 2006 [32]	Tanzania	2002–2003	Children (32,155)	1–35 months	No qualitative data.	*P. falciparum*	Microscopy	NS
Shankar et al., 2000 [33]	Papua New Guinea	1995	Children (274)	6–60 months	No differences were observed between groups for parasite prevalence. There was a tendency toward a higher prevalence of *P. falciparum* malaria in the zinc group (42%) compared to the placebo group (29%) (*p* = 0.050).	*P. falciparum*/*P. vivax*/*P. malariae*	Microscopy	Inductively coupled plasma analysis
Veenemans et al., 2011 [34]	Tanzania	2008–2009	Children (612)	6–60 months	There was no evidence that multi-nutrients influenced the effect of zinc (or vice versa). Neither zinc nor multi-nutrients influenced malaria rates.	*P. falciparum*/other *Plasmodium* spp.	Microscopy/RDT	Inductively coupled plasma-mass spectrometry (Varian 820-MS)
Zeba et al., 2008 [35]	Burkina Faso	NS	Children (148)	6–72 months	A significant decrease in the prevalence of malaria in the supplemented group (34%) compared to the placebo group (3.5%) was observed (*p* < 0.001).	*P. falciparum*	Microscopy	NS

NS, not specified; N, number of participants investigated.

## Data Availability

All data relating to the present study are available in this manuscript and Supplementary Files.

## Supplementary Materials

The following supporting information can be downloaded at: https://www.mdpi.com/article/10.3390/nu15132855/s1: Supplementary Figure S1. The meta-analysis demonstrated no effect of zinc on the risk of *P. falciparum* parasitaemia compared to placebo. Abbreviation: CI, confidence interval. Supplementary Figure S2. The meta-analysis demonstrated no significant effect of zinc plus other micronutrients on the risk of *P. falciparum* parasitaemia compared with placebo. Abbreviation: CI, confidence interval. Supplementary Figure S3. The meta-analysis demonstrated no significant effect of zinc plus other micronutrients on the risk of malaria parasitaemia compared with zinc alone. Abbreviation: CI, confidence interval. Supplementary Figure S4. The leave-one-out meta-analysis demonstrated no significant effect of zinc on the risk of malaria parasitaemia compared with placebo in all rerun analyses (*p* > 0.05). Abbreviation: CI, confidence interval. Supplementary Figure S5. The leave-one-out meta-analysis exhibited no substantial effect of zinc supplementation on the risk of malaria parasitaemia in every rerun analysis (*p* > 0.05). Abbreviation: CI, confidence interval. Supplementary Figure S6. The leave-one-out meta-analysis exhibited significant effects of zinc plus other micronutrients on the risk of malaria parasitaemia compared with placebo when the meta-analysis was rerun after removing each of the two studies (*p* < 0.05). Abbreviation: CI, confidence interval. Supplementary Figure S7. The leave-one-out meta-analysis showed no significant effect of zinc plus other micronutrients on the risk of malaria parasitaemia compared with placebo in every rerun analysis (*p* > 0.05). Abbreviation: CI, confidence interval. Supplementary Figure S8. The leave-one-out meta-analysis showed significant effects of zinc plus other micronutrients on the risk of malaria parasitaemia compared with zinc alone after removing and rerunning the analysis (*p* < 0.05). Abbreviation: CI, confidence interval. Supplementary Figure S9. The leave-one-out meta-analysis showed no significant effect of zinc plus other micronutrients on the risk of malaria parasitaemia compared with zinc alone in every rerun analysis (*p* > 0.05). Abbreviation: CI, confidence interval. Table S1. Search terms, Table S2. Details of the included studies, Table S3. Risk of bias.
