# Supplementary material for: Effects of Daily Zinc Alone or in Combination with Other Nutrient Supplements on the Risk of Malaria Parasitaemia: A Systematic Review and Meta-Analysis of Randomised Controlled Trials
Source: Nutrients. 2023 Jun 23;15(13):2855. doi: 10.3390/nu15132855 (PMC10346149; doi:10.3390/nu15132855)
Supplement: Supplementary file 1 [file nutrients-15-02855-s001.zip › Table S1. Search terms.pdf]

Effects of Daily Zinc Alone or in Combination with Other Nutrient Supplements on the Risk of Malaria Parasitaemia: A Systematic Review and Meta-Analysis of Randomised Controlled Trials

Manas Kotepui[1†*], Polrat Wilairatana[2†*], Wanida Mala[1], Kwuntida Uthaisar Kotepui[1], Frederick Ramirez Masangkay[3], Kinley Wangdi[4]

[1] Medical Technology, School of Allied Health Sciences, Walailak University, Tha Sala, Nakhon Si Thammarat, Thailand

[2] Department of Clinical Tropical Medicine, Faculty of Tropical Medicine, Mahidol University, Bangkok, Thailand

[3] Department of Medical Technology, University of Santo Tomas, Manila, Philippines

[4] Department of Global Health, National Centre for Epidemiology and Population Health, College of Health and Medicine, Australian National University, Canberra, Acton, ACT 2601, Australia

[*]Corresponding author

[†] These authors contributed equally to this work

Manas Kotepui manas.ko@wu.ac.th, Tel+ :.66954392469

Polrat Wilairatana: polrat.wil@mahidol.ac.th

Wanida Mala: wanida.ma@wu.ac.th

Kwuntida Uthaisar Kotepui: kwuntida.ut@wu.ac.th

Frederick Ramirez Masangkay: frederick_masangkay2002@yahoo.com

Kinley Wangdi: kinley.wangdi@anu.edu.au

Table S1. Search terms

General keywords

zinc AND (malaria OR plasmodium OR “Plasmodium Infection“ OR “Remittent Fever“ OR “Marsh Fever“ OR Paludism) AND (“Clinical Trials“ OR “Randomized Trials” OR “Randomized Controlled Trials” OR “Controlled Clinical Trials”)

PubMed 6 May 2023

No.	Key concept	Search terms	Results
1.	Zinc	zinc[tw] OR zinc [Mesh]	178,232
2.	Malaria	malaria[tw] OR plasmodium[tw] OR “Infections, Plasmodium“[Mesh] OR “Infection, Plasmodium“[Mesh] OR “Plasmodium Infection“[Mesh] OR “Remittent Fever“[Mesh] OR “Fever, Remittent“[Mesh] “Marsh Fever“[Mesh] OR “Fever, Marsh“[Mesh] OR Paludism[Mesh]	74,058
3.	Clinical trials	“Clinical Trials“[tw] OR “Randomized Trials” [tw] OR “Randomized Controlled Trials” [tw] OR “Controlled Clinical Trials” [tw] OR “Clinical Trials“[Mesh] OR “Randomized Controlled Trials”[Mesh]	687,359
3.	1 AND 2 AND 3	(zinc[tw] OR zinc [Mesh]) AND (malaria[tw] OR plasmodium[tw] OR “Infections, Plasmodium“[Mesh] OR “Infection, Plasmodium“[Mesh] OR “Plasmodium Infection“[Mesh] OR “Remittent Fever“[Mesh] OR “Fever, Remittent“[Mesh] “Marsh Fever“[Mesh] OR “Fever, Marsh“[Mesh] OR Paludism[Mesh]) AND (“Clinical Trials“[tw] OR “Randomized Trials” [tw] OR “Randomized Controlled Trials” [tw] OR “Controlled Clinical Trials” [tw] OR “Clinical Trials“[Mesh] OR “Randomized Controlled Trials”[Mesh])	17

Embase 6 May 2023

No.	Key concept	Search terms	Results
1.	Zinc	zinc:ti,ab,kw,de OR zinc/exp	294,567
2.	Malaria	malaria:ti,ab,kw,de OR plasmodium:ti,ab,kw,de OR ‘Remittent Fever’:ti,ab,kw,de OR ‘Marsh Fever’:ti,ab,kw,de OR Paludism:ti,ab,kw,de OR malaria/exp	155,458
3.	Clinical trials	“Clinical Trials“:ti,ab,kw,de OR “Randomized Trials”:ti,ab,kw,de OR “Randomized Controlled Trials”:ti,ab,kw,de OR “Controlled Clinical	845,731

		Trials”:ti,ab,kw,de OR “Clinical Trials“/exp OR “Randomized Controlled Trials”/exp	
4..	1 AND 2 AND 3	(zinc:ti,ab,kw,de OR zinc/exp) AND (malaria:ti,ab,kw,de OR plasmodium:ti,ab,kw,de OR ‘Remittent Fever’:ti,ab,kw,de OR ‘Marsh Fever’:ti,ab,kw,de OR Paludism:ti,ab,kw,de OR malaria/exp) AND (“Clinical Trials“:ti,ab,kw,de OR “Randomized Trials”:ti,ab,kw,de OR “Randomized Controlled Trials”:ti,ab,kw,de OR “Controlled Clinical Trials”:ti,ab,kw,de OR “Clinical Trials“/exp OR “Randomized Controlled Trials”/exp)	54

Scopus 6 May 2023

No.	Key concept	Search terms	Results
1.	Zinc	TITLE-ABS-KEY (zinc)	678,133
2.	Malaria	TITLE-ABS-KEY (malaria OR plasmodium OR "plasmodium infection" OR "remittent fever" OR "marsh fever" OR paludism)	156,232
3.	Clinical trials	TITLE-ABS-KEY ("Clinical Trials" OR "Randomized Trials" OR "Randomized Controlled Trials" OR "Controlled Clinical Trials")	2,192,383
4.	1 AND 2 AND 3	(TITLE-ABS-KEY (zinc)) AND (TITLE-ABS-KEY (malaria OR plasmodium OR "plasmodium infection" OR "remittent fever" OR "marsh fever" OR paludism)) AND TITLE-ABS-KEY ("Clinical Trials" OR "Randomized Trials" OR "Randomized Controlled Trials" OR "Controlled Clinical Trials")	169

MEDLINE 6 May 2023

No.	Key concept	Search terms	Results
1.	Zinc AND Malaria AND Clinical trials	zinc AND (malaria OR plasmodium OR “Plasmodium Infection“ OR “Remittent Fever“ OR “Marsh Fever“ OR Paludism) AND (“Clinical Trials“ OR “Randomized Trials” OR “Randomized Controlled Trials” OR “Controlled Clinical Trials”)	28

Ovid 6 May 2023

No.	Key concept	Search terms	Results
1.	Zinc AND Malaria AND Clinical trials	zinc AND (malaria OR plasmodium OR “Plasmodium Infection“ OR “Remittent Fever“ OR “Marsh Fever“ OR Paludism) AND (“Clinical Trials“ OR “Randomized Trials” OR “Randomized Controlled Trials” OR “Controlled Clinical Trials”) {No Related Terms}	110

		limit to (ovid full text available and articles with abstracts and original articles)	

ProQuest 6 May 2023

No.	Key concept	Search terms	Results
1.	Zinc AND Malaria AND Clinical trials	zinc AND (malaria OR plasmodium OR “Plasmodium Infection“ OR “Remittent Fever“ OR “Marsh Fever“ OR Paludism) AND (“Clinical Trials“ OR “Randomized Trials” OR “Randomized Controlled Trials” OR “Controlled Clinical Trials”)	961
