# Supplementary figures and images for: Effects of Daily Zinc Alone or in Combination with Other Nutrient Supplements on the Risk of Malaria Parasitaemia: A Systematic Review and Meta-Analysis of Randomised Controlled Trials
Source: Nutrients. 2023 Jun 23;15(13):2855. doi: 10.3390/nu15132855 (PMC10346149; doi:10.3390/nu15132855)

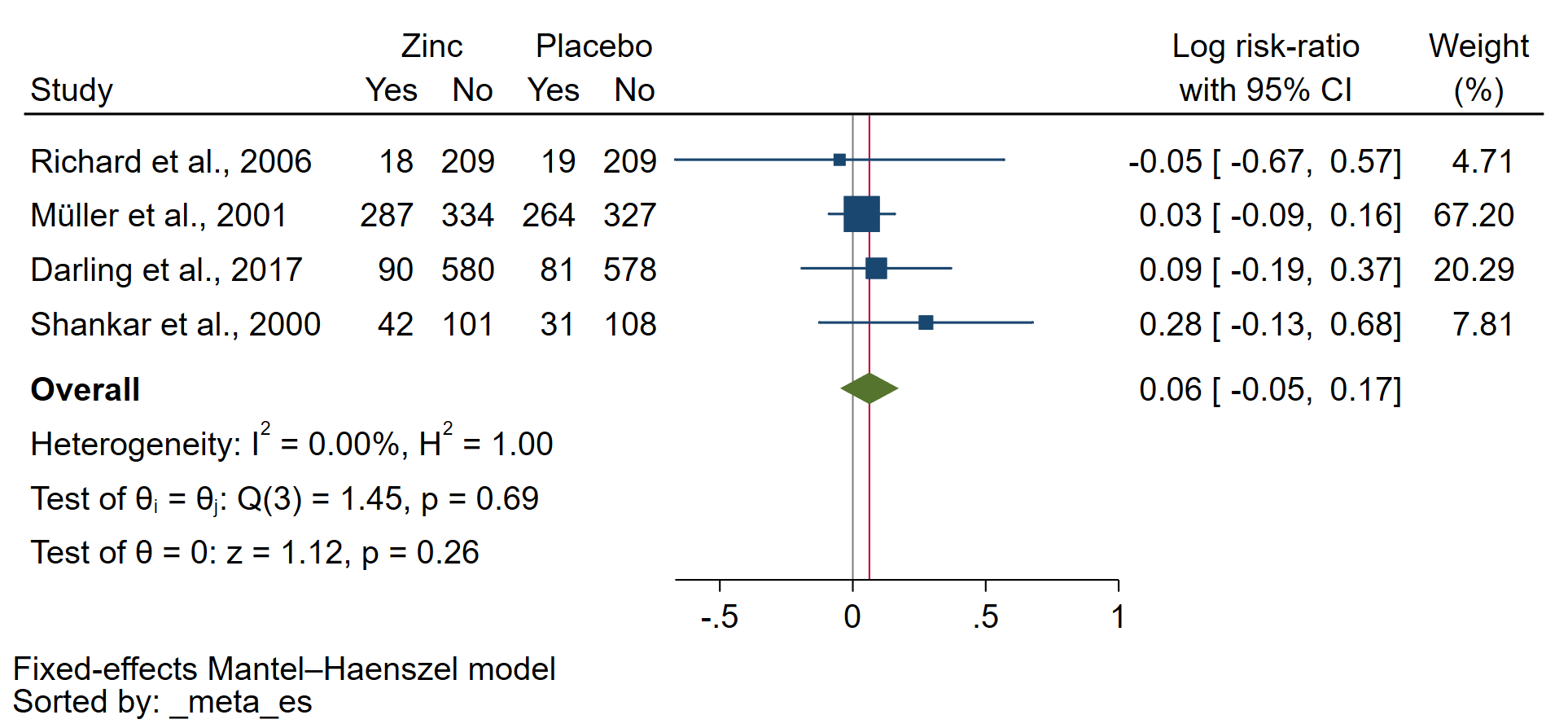

Supplement: Supplementary file 1 [file nutrients-15-02855-s001.zip › Supplementary Figure S1. Zinc vs placebo_Pf.tif]

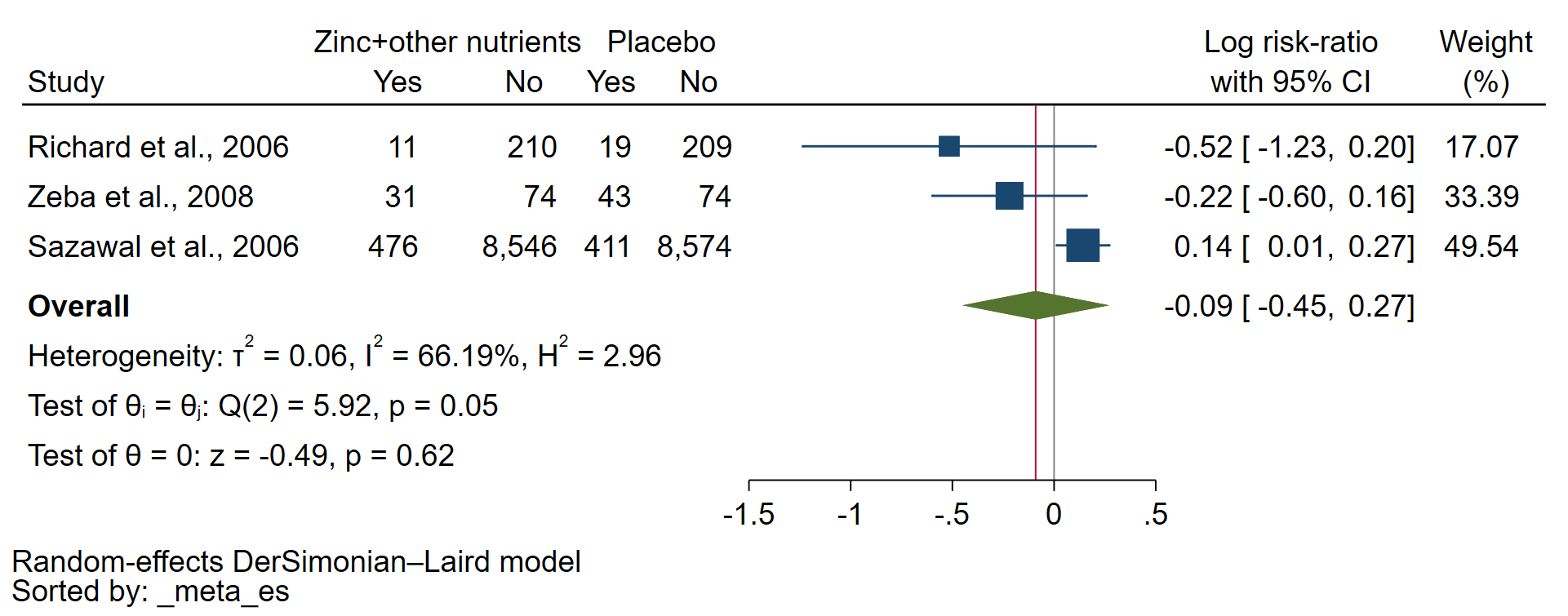

Supplement: Supplementary file 1 [file nutrients-15-02855-s001.zip › Supplementary Figure S2. Zinc+others vs placebo_Pf.tif]

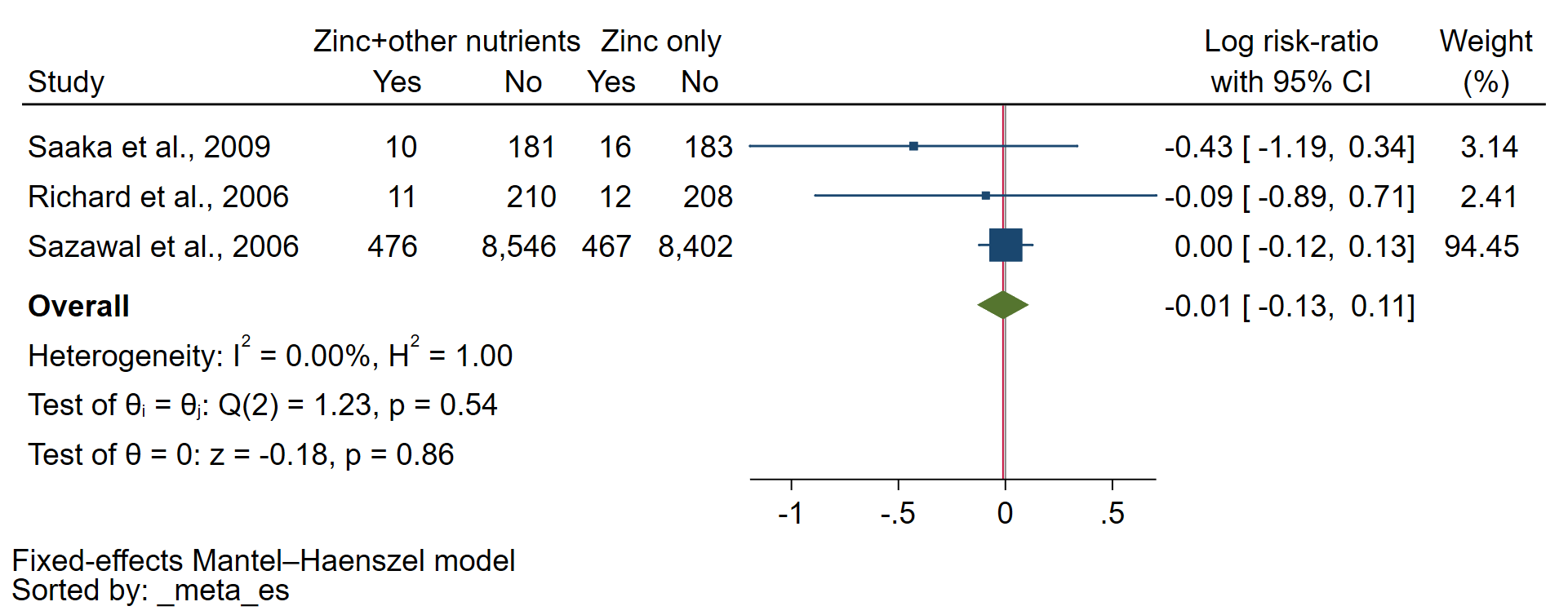

Supplement: Supplementary file 1 [file nutrients-15-02855-s001.zip › Supplementary Figure S3. Zinc+others vs zinc only_Pf.tif]

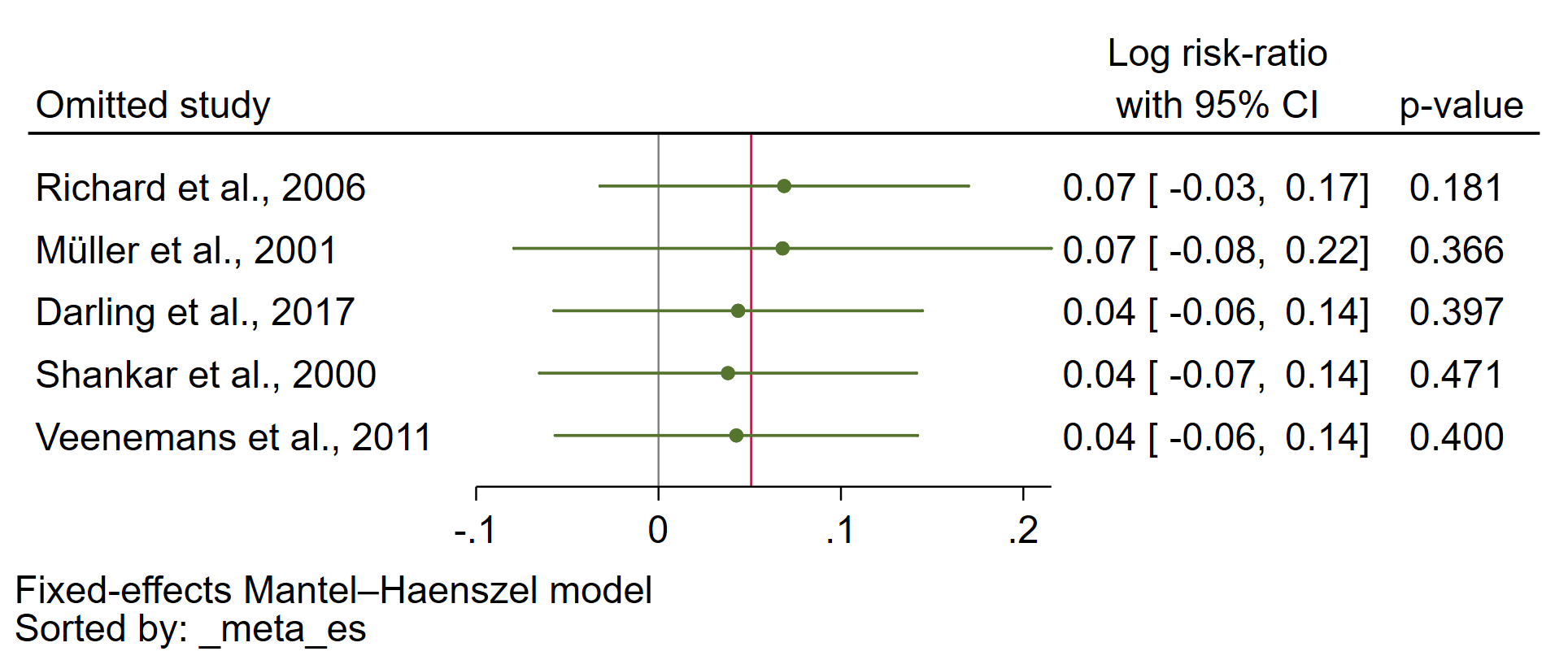

Supplement: Supplementary file 1 [file nutrients-15-02855-s001.zip › Supplementary Figure S4. Zinc vs placebo_All species_leaveoneout.tif]

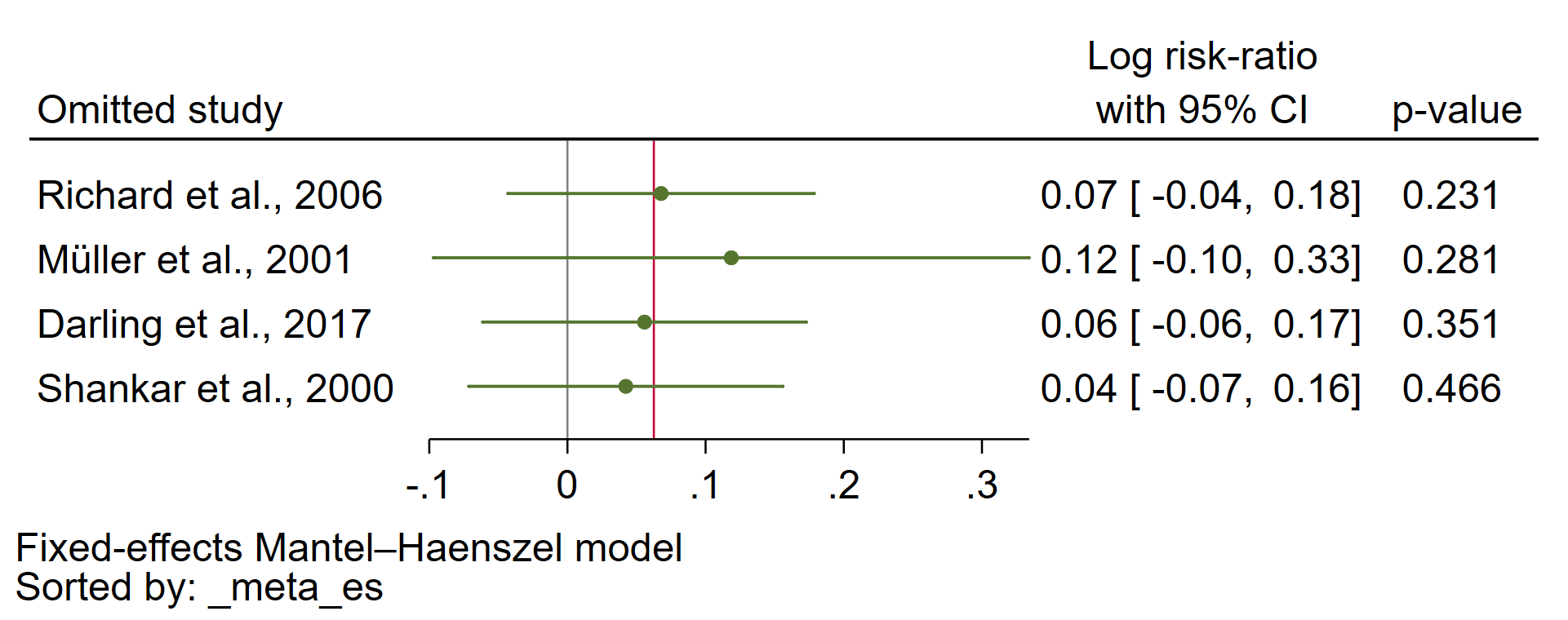

Supplement: Supplementary file 1 [file nutrients-15-02855-s001.zip › Supplementary Figure S5. Zinc vs placebo_Pf_leaveoneout.tif]

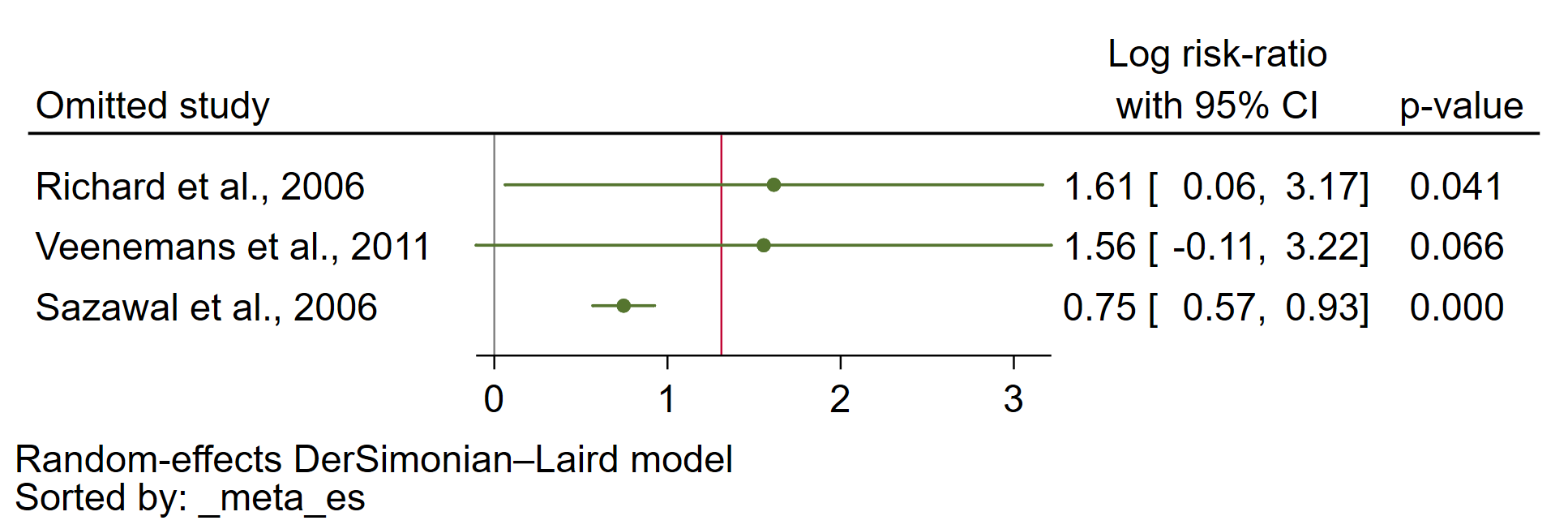

Supplement: Supplementary file 1 [file nutrients-15-02855-s001.zip › Supplementary Figure S6. Zinc+others vs placebo_All species_leaveoneout.tif]

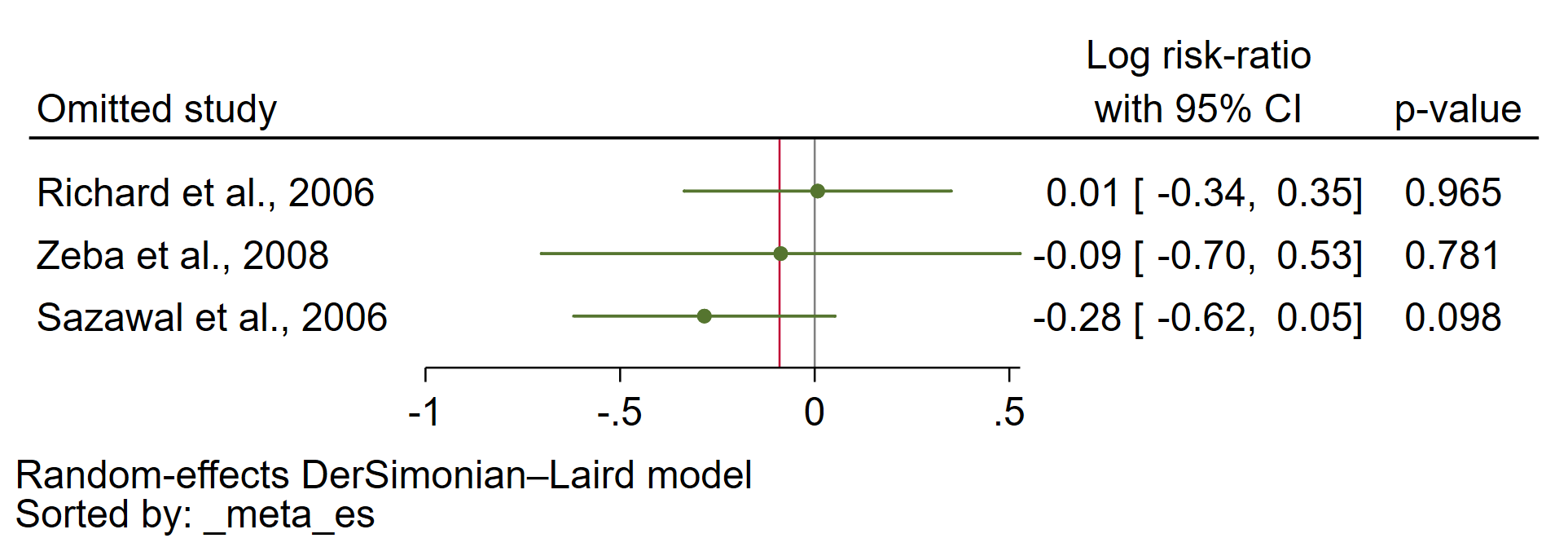

Supplement: Supplementary file 1 [file nutrients-15-02855-s001.zip › Supplementary Figure S7. Zinc+others vs placebo_Pf_leaveoneout.tif]

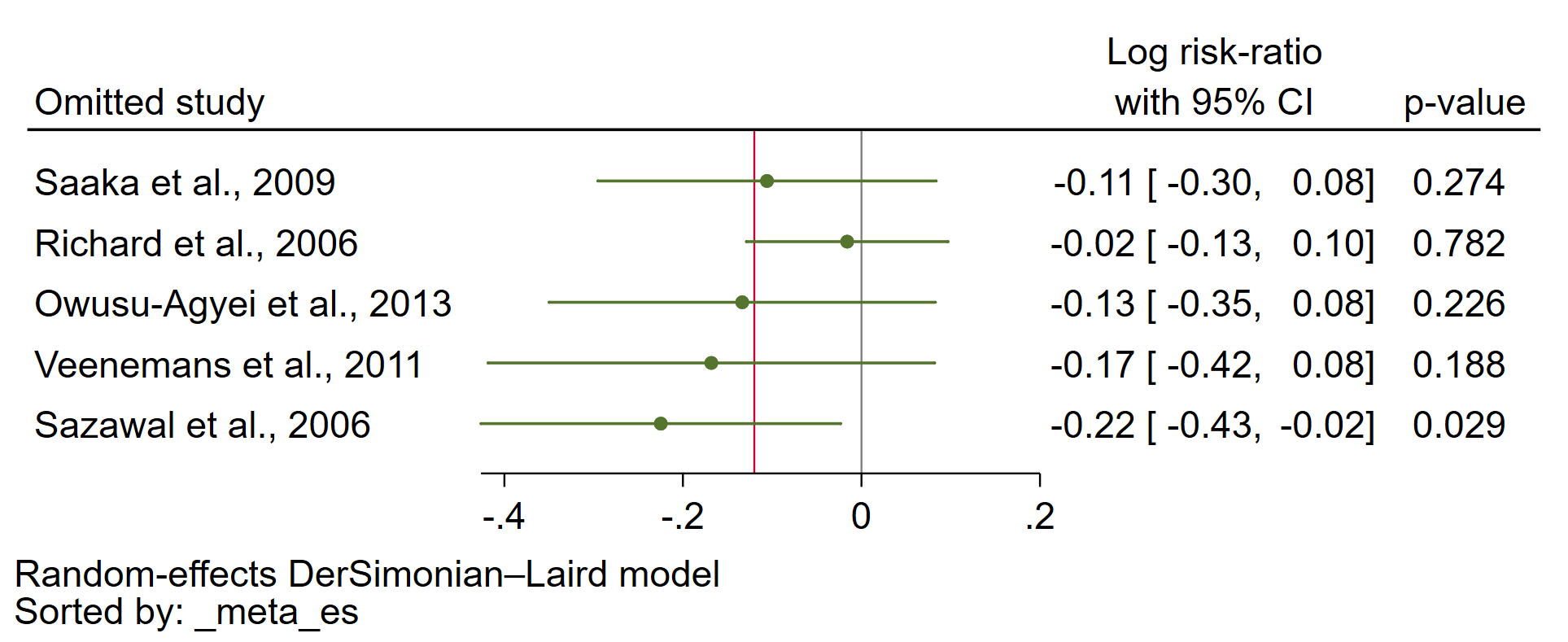

Supplement: Supplementary file 1 [file nutrients-15-02855-s001.zip › Supplementary Figure S8. Zinc+others vs zinc only_All species_leaveoneout.tif]

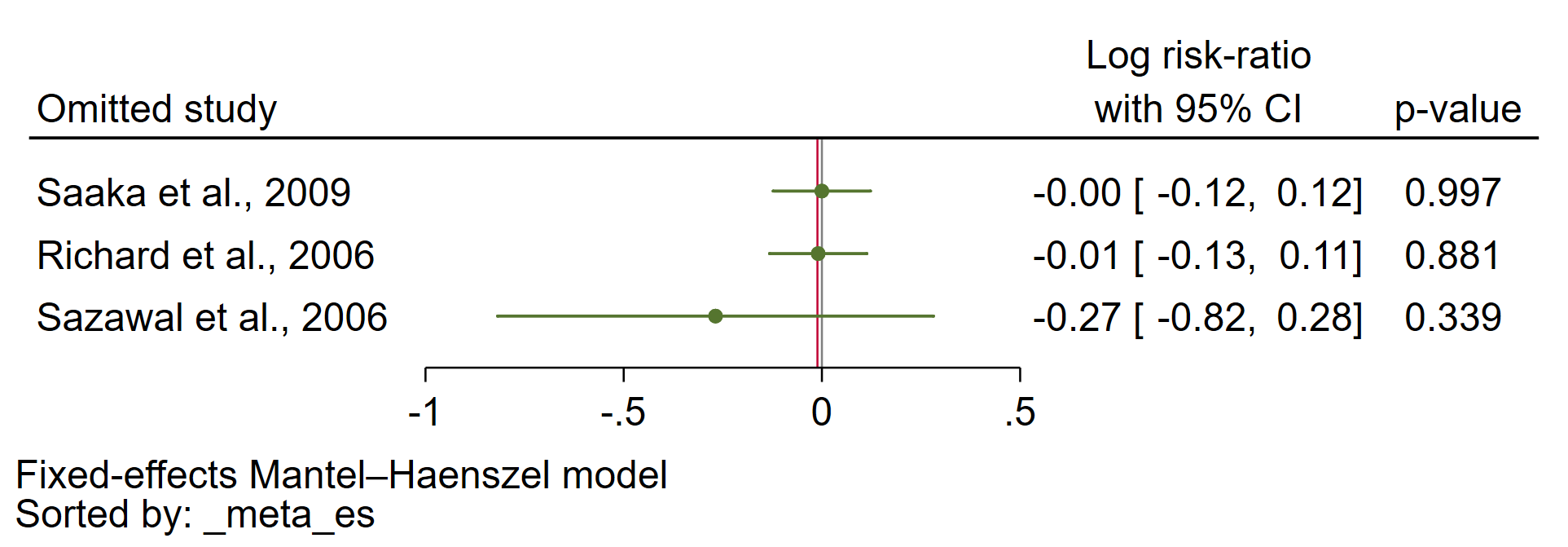

Supplement: Supplementary file 1 [file nutrients-15-02855-s001.zip › Supplementary Figure S9. Zinc+others vs zinc only_Pf_leaveoneout.tif]
